# Atrogin-1 Deficiency Leads to Myopathy and Heart Failure in Zebrafish

**DOI:** 10.3390/ijms17020187

**Published:** 2016-01-30

**Authors:** Anja Bühler, Monika Kustermann, Tiziana Bummer, Wolfgang Rottbauer, Marco Sandri, Steffen Just

**Affiliations:** 1 Molecular Cardiology, University of Ulm, 89081 Ulm, Germany; anja.buehler@uniklinik-ulm.de (A.B.); monika.forster@uni-ulm.de (M.K.); tiziana.bummer@uni-ulm.de (T.B.); 2 Department of Internal Medicine II, University of Ulm, 89081 Ulm, Germany; wolfgang.rottbauer@uniklinik-ulm.de; 3 Department of Biomedical Sciences, University of Padova, 35129 Padova, Italy; marco.sandri@unipd.it

**Keywords:** Atrogin-1, autophagy, zebrafish, myopathy, heart failure

## Abstract

Orchestrated protein synthesis and degradation is fundamental for proper cell function. In muscle, impairment of proteostasis often leads to severe cellular defects finally interfering with contractile function. Here, we analyze for the first time the role of Atrogin-1, a muscle-specific E3 ubiquitin ligase known to be involved in the regulation of protein degradation via the ubiquitin proteasome and the autophagy/lysosome systems, in the *in vivo* model system zebrafish (*Danio rerio*). We found that targeted inactivation of zebrafish Atrogin-1 leads to progressive impairment of heart and skeletal muscle function and disruption of muscle structure without affecting early cardiogenesis and skeletal muscle development. Autophagy is severely impaired in Atrogin-1-deficient zebrafish embryos resulting in the disturbance of the cytoarchitecture of cardiomyocytes and skeletal muscle cells. These observations are consistent with molecular and ultrastructural findings in an Atrogin-1 knockout mouse and demonstrate that the zebrafish is a suitable vertebrate model to study the molecular mechanisms of Atrogin-1-mediated autophagic muscle pathologies and to screen for novel therapeutically active substances in high-throughput *in vivo* small compound screens (SCS).

## 1. Introduction

Protein homeostasis (proteostasis) describes the sum of processes involved in protein biogenesis, folding, modification, trafficking, assembly, and degradation within and also outside of the cell [1]. In this context, protein degradation as a mechanism to control protein quality and quantity in the cell, is predominately accomplished by two highly effective proteolytic machineries, the autophagy/lysosome system and the Ubiquitin proteasome degradation system (UPS) [2]. Recent studies in *in vitro* systems and animal models unraveled a crucial role of lysosomal and proteasomal insufficiency for the onset and progression of heart and skeletal muscle as well as neurodegenerative diseases [3,4,5,6,7]. The evolutionary highly conserved macroautophagy machinery (from here on referred to as autophagy) is not only important for the turnover of organelles and cytoplasmic components but also proteins [2,8]. The autophagy pathway is subdivided into three major stages (1) membrane commitment; (2) membrane expansion to form the autophagosome; and (3) autophagosome docking and fusion with lysosomes for cargo degradation [1,6,9]. The UPS is known to hydrolyze intracellular proteins into small peptides, thereby controlling protein turnover and clearance of native or misfolded and (poly-)ubiquitinated proteins [3,10]. Protein ubiquitination is catalyzed by the sequential function of three different enzymes, (1) the ubiquitin activating enzyme (E1), the ubiquitin conjugating enzyme (E2), and the ubiquitin ligase (E3), the latter one mediating substrate-specificity of protein degradation through the UPS [1]. Very recently, Atrogin-1 (MAFbx), a muscle-specific E3 ubiquitin ligase, was identified to be critically involved in targeting important muscular signaling proteins for degradation and when defective leads to cardiomyopathy and skeletal muscle dysfunction [11,12,13,14].

To further elucidate the *in vivo* role of Atrogin-1 in the vertebrate heart and skeletal muscle, we generated Atrogin-1-deficient zebrafish. We show that targeted ablation of Atrogin-1 leads to reduced systolic cardiac force and bradycardia, phenotypic hallmarks of human heart failure. Additionally, skeletal muscle function was compromised by Atrogin-1 ablation in the zebrafish embryo, suggesting that, similar to the situation in mammals, Atrogin-1 might play a crucial role in orchestrating protein degradation in the zebrafish heart and skeletal muscle. Indeed, we find the autophagy/lysosome system significantly impaired in Atrogin-1 morphants. Accordingly, we identified severe autophagy-associated ultrastructural alterations including sarcomeric disassembly, dysmorphic mitochondria, and vesicular bodies in Atrogin-1-deficient cardiomyocytes and skeletal muscle cells.

In summary, by targeted gene inactivation, we demonstrate an important role of Atrogin-1 in regulating protein degradation in the embryonic zebrafish heart and skeletal muscle and thereby present a straight-forward *in vivo* model to further dissect the molecular pathogenetic mechanisms associated with autophagy.

## 2. Results

### 2.1. Zebrafish Atrogin-1 Localizes Cytoplasmatically in Cardiac and Skeletal Muscle Cells

**Figure 1:**
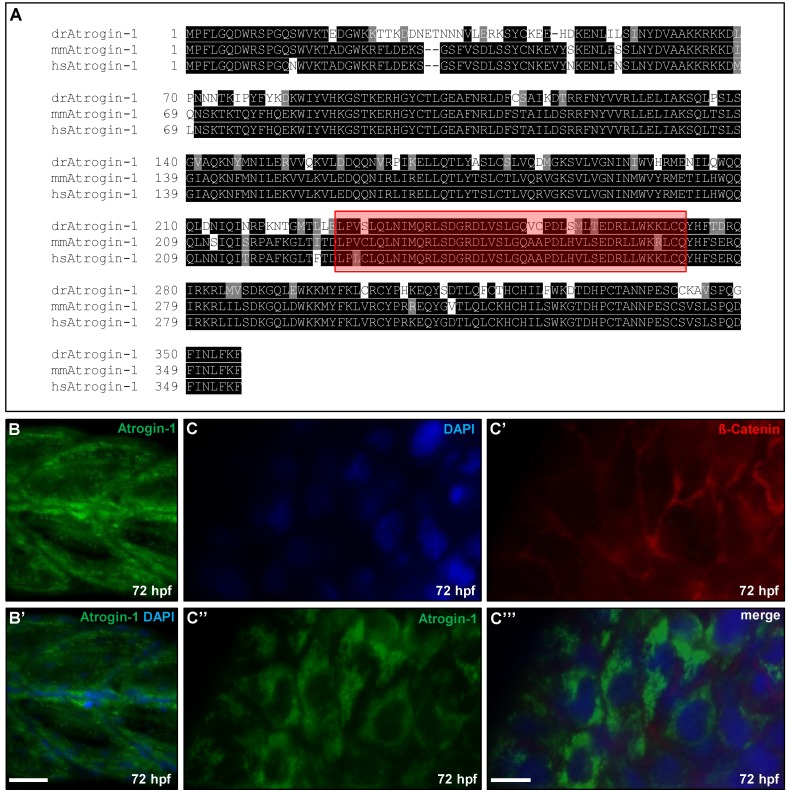
Atrogin-1 is conserved between humans, mice, and zebrafish and zebrafish Atrogin-1 is expressed in the embryonic skeletal muscle and heart. (**A**) Protein alignment of *Danio rerio* (*dr*), *Mus musculus* (*mm*) and *Homo sapiens* (*hs*) Atrogin-1, identical amino acids are shaded in black, similar amino acids in grey. Conserved F-box domain is depicted in red; (**B**,**B’**) Immunofluorescent staining against Atrogin-1 on paraffin sections of zebrafish skeletal muscle at 72 hpf, nuclei are counterstained with DAPI, scale bar = 25 µm; (**C**-**C’’’**) Immunofluorescent staining against Atrogin-1 (green) and ß-Catenin (red) on dissected zebrafish hearts at 72 hpf, nuclei are counterstained with DAPI, scale bar = 10 µm.

To study the *in vivo* role of Atrogin-1 (FbxO32) in zebrafish heart and skeletal muscle development and function, we first identified the zebrafish orthologous sequence by BLAST analysis of the mouse Atrogin-1 sequence against the NCBI zebrafish protein database and analyzed amino acid conservation between zebrafish and mouse or human Atrogin-1. Zebrafish Atrogin-1 protein is evolutionarily conserved, showing 75.1% amino acid identity with mouse Atrogin-1 and 75.7% with human Atrogin-1 (Figure 1A). As in mouse or human Atrogin-1, the zebrafish orthologue contains a highly conserved F-box domain that is known to convey its E3 ubiquitin ligase activity (Figure 1A highlighted in red, zebrafish 229–268 aa, mouse 228–267 aa) [15]

In mammals, Atrogin-1 is predominantly expressed in the heart and skeletal muscle cells [11]. In zebrafish, we found Atrogin-1 to be ubiquitously expressed at low levels, but with pronounced expression in muscle tissue at 72 h post fertilization (hpf) (Figure S1J, J’). Hence, to evaluate the subcellular localization of Atrogin-1 in zebrafish heart and skeletal muscle, we performed immunostainings of zebrafish skeletal muscle sections using an Atrogin-1-specific antibody. As revealed by co-immunostaining with the nuclear marker DAPI (4′,6-Diamidin-2-phenylindol), zebrafish Atrogin-1 protein localized mainly in the cytoplasm of zebrafish skeletal muscle cells at 72 hpf (Figure 1B,B’). Furthermore, by co-immunostaining of dissected embryonic zebrafish hearts (72 hpf) using anti-Atrogin-1 and anti-ß-Catenin antibodies, we found Atrogin-1, similar to the situation in skeletal muscle cells, localized mainly in the cytoplasm of zebrafish embryonic cardiomyocytes (Figure 1C–C’’’).

### 2.2. Inactivation of Atrogin-1 Leads to Myopathy and Heart Failure in Zebrafish Embryos

**Figure 2:**
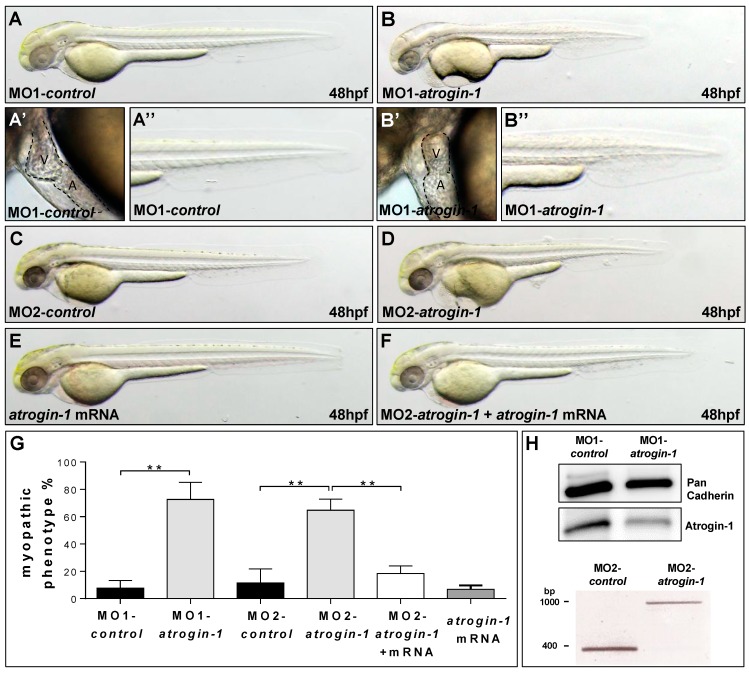
Knock-down of Atrogin-1 leads to defects in muscle and heart morphology. (**A**,**B**) Lateral view of embryos at 48 hpf injected either by 5 bp mismatch Start Morpholino (MO1-*control*, **A**) or Start Morpholino directed against atrogin-1 (MO1-*atrogin-1*, **B**); (**A’**,**A’’**–**B’**,**B’’**) Close-ups of heart and tail of injected embryos at 48 hpf; (**C**,**D**) Lateral view of embryos at 48 hpf either injected by a 5 bp mismatch Morpholino targeting a splice donor site of Atrogin-1 as a control (MO2-*control*) or a Splice Morpholino (MO2-*atrogin-1*); (**E**,**F**) Lateral view of embryos at 48 hpf. Control injection with *atrogin-1* mRNA (**E**); Rescue of the morphant phenotype by injecting *atrogin-1* mRNA and MO2-*atrogin-1* together (**F**); (**G**) Statistical analysis of affected embryos after MO1/2-*control*, MO1/2-*atrogin-1*, *atrogin-1* mRNA or MO2-*atrogin-1* and *atrogin-1* mRNA. Data represent means ± SD, student’s *t*-test, ** *p*-value < 0.0022; (**H**) Western Blot analysis using an Atrogin-1-specific antibody after MO1-*control* and MO1-*atrogin-1* injection, RT-PCR after injection of MO2-*control*, or MO2-*atrogin-1* showing specific effect of MO injection. Atrium (A) and ventricle (V).

Next, to investigate the role of Atrogin-1 in the zebrafish heart and skeletal muscle *in vivo*, we inactivated zebrafish Atrogin-1 by microinjection of two independent Morpholino-modified antisense oligonucleotides (MO) directed against either the translational start site (MO1-*atrogin-1*) or the splice-donor site of exon 1 (MO2-*atrogin-1*) into one-cell-stage wild-type zebrafish embryos. Injection of 5.4 ng of MO1-*atrogin-1* leads to a less organized muscle structure and the development of a pericardial edema in 72.7% ± 12.4% of injected wild-type embryos (*n* = 383, three independent experiments) (Figure 2B–B’’,G). However, embryos injected with the same amount of a corresponding five base pair mismatch Morpholino (MO1-*control*) (*n* = 321, three independent experiments) did not exhibit impaired cardiac and skeletal muscle morphology (Figure 2A–A’’,G). Injection of the second, independent splice site targeting MO (MO2-*atrogin-1*) led to similar heart and skeletal muscle defects as injection of MO1-*atrogin-1*. By injecting of 7.2 ng of MO2-*atrogin-1*, 64.7% ± 8.0% of injected embryos (*n* = 227, three independent experiments) show this pathology (Figure 2D), whereas injection of the corresponding five base pair mismatch Morpholino (MO2-*control*) (*n* = 248, three independent experiments) had no impact on the heart and skeletal muscle (Figure 2C).

To ensure that the used Morpholino-modified antisense oligonucleotides are indeed functional in knocking down Atrogin-1 *in vivo*, we performed Western Blot analyses and mRNA splicing assays in embryos injected with MO1-*atrogin-1* and MO2-*atrogin-1*, respectively. By immunoblot analysis of embryos injected with MO1-*atrogin-1* and MO1-*control*, we found a severe reduction (56%) of Atrogin-1 protein levels in MO1-*atrogin-1* injected embryos compared to control-injected embryos at 72 hpf, suggesting that the translation blocking Morpholino is functional *in vivo* (Figure 2H). Isolation of mRNA from MO2-*atrogin-1* injected embryos at 24 hpf and subsequent RT-PCR confirmed the proposed impact of this MO on *atrogin-1* mRNA splicing. As shown in Figure 2H, in MO2-*atrogin-1* injected embryos, one aberrant splice product that shows the integration of intron 1 resulting in the generation of a premature stop codon was detected. Additionally, the amount of wild-type *atrogin-1* mRNA was strongly reduced in the morphants (Figure 2H). Moreover, Western Blot analysis of 72 h old MO2-*atrogin-1* and MO2-*control* injected embryos revealed, similar to the situation in MO1-*atrogin-1* injected embryos, a severe reduction of Atrogin-1 protein levels (Figure S1C). To further prove specificity of the knock-down, we ectopically expressed Myc-tagged wild-type *atrogin-1* mRNA in Atrogin-1 morphant embryos and evaluated the heart and skeletal muscle phenotype in these double-injected embryos. Whereas 64.7% ± 8.0% of MO2-*atrogin-1* and KCl co-injected embryos developed a heart and skeletal muscle defect, only 18.4% ± 5.5% of the embryos injected with MO2-*atrogin-1* and *atrogin-1* mRNA (*n* = 187, three independent experiments) exhibited these pathologies (Figure 2F,G; Figure S1B). Injection of the same amount of *atrogin-1* mRNA into wild-type zebrafish embryos (*n* = 193, three independent experiments) had no effect on heart and skeletal muscle function and morphology (Figure 2E,G; Figure S1B). These findings indicate that the effects induced by MOs targeting zebrafish Atrogin-1 were specific and not due to off-target effects.

**Figure 3:**
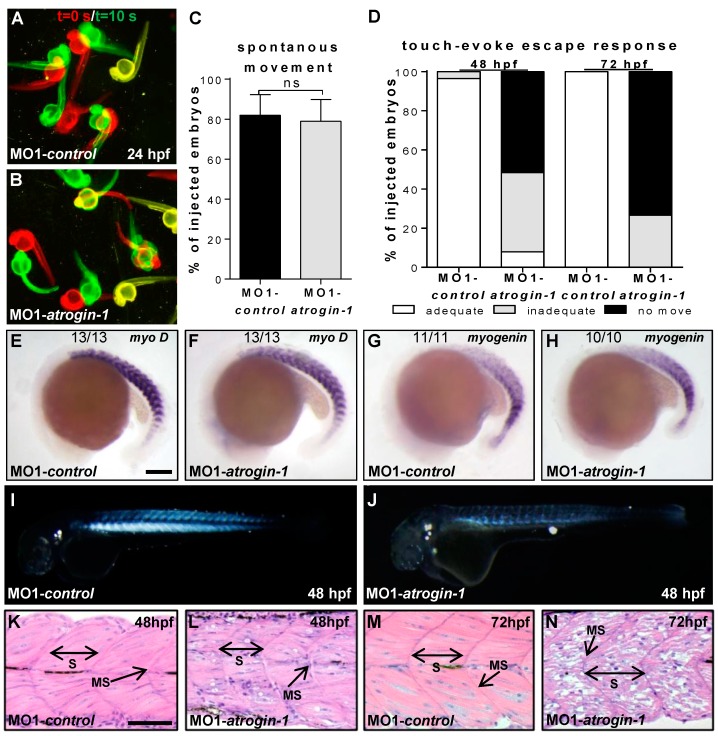
Myopathic phenotype in Atrogin-1 knock-down embryos is progressive. (**A**,**B**) Spontaneous movement assay with false-colored superimposed overviews of 24 hpf control (**A**) and Atrogin-1 morphants (**B**); red pictures = 0 s; green pictures = 10 s; yellow = merge; (**C**) Statistical analysis of spontaneous movement assay, data represent means ± SD, student’s *t*-test *p*-value < 0.27, ns = not significant; (**D**) Quantification of touch-evoke flight response of 48 hpf and 72 hpf MO1-*control* and MO1-*atrogin-1* injected embryos; (**E**–**H**) Lateral view of whole-mount antisense RNA *in situ* hybridization against *myoD* at 18-somite stage (**E**,**F**) and against *myogenin* at the 16-somite stage (**G**,**H**) scale bar = 100 µm. Expression of *MyoD* and *myogenin* is unaltered in Atrogin-1 morphants; (**I**,**J**) Lateral view of MO1-*control* (**I**) and MO1-*atrogin-1* injected embryos (**J**) showing birefringence at 48 hpf; (**K**–**N**) H&E-stained sagittal histological sections of skeletal muscle at 48 and 72 hpf. S = somite, MS = myoseptum, arrow indicate somite borders, scale bar = 50 µm.

To describe the heart and skeletal muscle pathologies in more detail, we performed several functional and structural analyses. At 24 hpf, similar to control-injected embryos (81.9% ± 10.4%, *n* = 69, three independent experiments), Atrogin-1 morphants showed regular spontaneous movements (78.9% ± 11.0%, *n* = 62, three independent experiments; Figure 3A–C). By contrast, at 48 hpf, 92.0% of Atrogin-1 morphants started to exhibit the impairment of motility after touch stimulation (*n* = 35, three independent experiments) (Figure 3D, Video S1), whereas 96.4% ± 7.2% of control embryos (*n* = 35, three independent experiments) execute a fast and powerful flight response after tactile stimulation (Figure 3D, Video S2). At 72 hpf, 26.7% ± 11.6% of Atrogin-1-deficient embryos demonstrated an inadequate touch-evoke flight response whereas 73.3% ± 11.6% of Atrogin-1 morphants were paralyzed (*n* = 15, three independent experiments, Figure 3D). Embryos injected with the control morpholino MO1-*control* showed no impaired motility at 72 hpf (*n* = 16, three independent experiments, Figure 3D). These findings demonstrate that Atrogin-1 deficiency leads to the loss of skeletal muscle function over time. Interestingly, the expression of essential myogenic markers such as *myoD* and *myogenin* (*myoG*) at the 18 and 16 somite stages, respectively, was unaffected by the loss of Atrogin-1, suggesting the regular specification to the myogenic lineage (Figure 3E–H).

Next, to investigate whether impaired motility of Atrogin-1 morphants beginning at 48 hpf was caused by structural alterations of the muscle, we first assessed muscle architecture of Atrogin-1-deficient embryos by a birefringence assay. The high structural organization of muscle tissue enables the polarization of light resulting in a strong birefringence signal after illumination. By contrast, disruption or disorganization of the muscle tissue leads to reduction or loss of the birefringence signal thereby visualizing muscle damage in a non-invasive manner [16]. Interestingly, birefringence signal intensity from the skeletal muscle was severely reduced in Atrogin-1-deficient embryos at 48 hpf, whereas muscle structure was unaffected in embryos injected with control Morpholino (Figure 3I,J). At 72 hpf, the birefringence signal was almost completely abolished compared to control embryos (Figure S1D,E), implying the progressive and significant loss of skeletal muscle organization and structure. To further substantiate this finding, we performed histological sections from Atrogin-1-deficient and control skeletal muscle tissue at 48 and 72 hpf. At 48 hpf, myofilaments in Atrogin-1 morphants appeared less organized as well as pinched out (Figure 3L) compared to control injected embryos (Figure 3K). At 72 hpf, this structural phenotype of Atrogin-1 morphants further worsens. Atrogin-1-deficient skeletal muscle tissue was unorganized and vacuolated at this developmental time point (Figure 3N), implying that the observed paralysis of Atrogin-1 morphant embryos is due to disrupted skeletal muscle organization.

**Figure 4:**
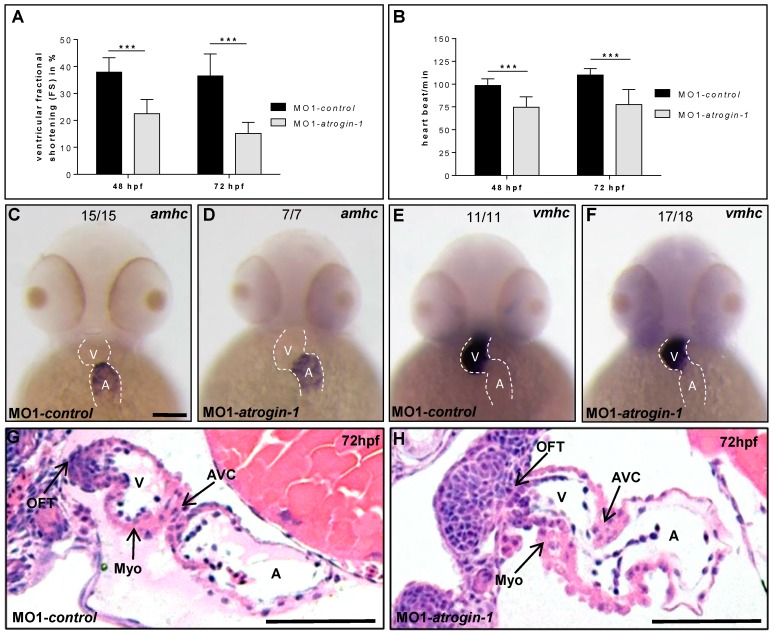
Impaired heart function in Atrogin-1-deficient embryos. (**A**) Fractional shortening (FS) measurements of the ventricular chambers of Atrogin-1 morphants and embryos injected with control Morpholino at 48 and 72 hpf; (**B**) Heart beat per minute of MO1-*control* compared to MO1-*atrogin-1* injected embryos measured at 48 and 72 hpf. Data represent means ± SD, student´s *t*-test, *** p< 0.0001 (**A**,**B**); (**C**–**F**) Whole-mount antisense RNA *in situ* hybridization against *atrial myosin heavy chain* (*amhc*) and *ventricular myosin heavy chain* (*vmhc*) demonstrates unaltered mRNA expression in Atrogin-1 morphants at 48 hpf, scale bar = 50 µm; (**G**,**H**) H&E-stained sagittal histological sections shows normal heart morphology in MO1-*control* and MO1-*atrogin-1* morphant hearts at 72 hpf. Atrium (A), ventricle (V), myocardium (Myo), atrio-ventricular canal (AVC) and outflow tract (OFT), scale bar = 50 µm.

Besides the loss of skeletal muscle function, cardiac contractility was also impaired in Atrogin-1 morphant embryos. At 24 hpf, similar to control embryos, Atrogin-1 morphant hearts exhibited strong and regular peristaltic contractions. By contrast, contractile force of the ventricular chamber of Atrogin-1-deficient hearts started to decline at 48 hpf (Fractional shortening (FS): 21.5 ± 5.1, *n* = 15, three independent experiments), whereas the injection of control Morpholino did not affect contractility (FS: 37.9 ± 5.2, *n* = 14, three independent experiments) (Figure 4A). At 72 hpf, contractile dysfunction in Atrogin-1 morphants worsened even further compared to control-injected embryos (FS Atrogin-1 morphants: 15.2 ± 3.9, *n* = 15, three independent experiments *vs.* FS control: 36.5 ± 7.3, *n* = 14, three independent experiments) (Figure 4A; Video S3 (MO1*-atrogin-1*) and S4 (MO1-*control*)), resulting in reduced blood flow through the vascular bed and blood congestion at the cardiac inflow tract.

In addition to reduced contractile force, Atrogin-1 morphants displayed significantly reduced heart rates (HR) at 48 hpf (HR Atrogin-1 morphants: 74.8 ± 10.9, *n* = 15, three independent experiments) and 72 hpf (HR Atrogin-1 morphants: 77.6 ± 16.2, *n* = 15, three independent experiments) compared to control-injected embryos (HR control 48 hpf: 98.4 ± 7.2, *n* = 14, three independent experiments and HR control 72 hpf: 110.1 ± 6.7, *n* = 14, three independent experiments) (Figure 4B). Furthermore, whole-mount RNA *in situ* hybridization against *atrial myosin heavy chain* (*amhc; myh6*) and *ventricular myosin heavy chain* (*vmhc*) at 48 hpf demonstrated an unaltered expression of these essential cardiac differentiation markers in Atrogin-1 morphant embryos (Figure 4C–F).

Subsequently, to assess whether loss of Atrogin-1 in the zebrafish leads to structural alterations of the heart, we performed sagittal histological sections through Atrogin-1 morphant and control hearts at 72 hpf. As shown in Figure 4G,H, we found that cardiac chambers, similar to the situation in control-injected hearts, are well defined in Atrogin-1-deficient hearts, with atrium and ventricle separated by the atrio-ventricular canal. In addition, endocardial and myocardial cell layers of both heart chambers had developed regularly with a multi-layered ventricular myocardium (Figure 4G,H).

### 2.3. The Autophagy/Lysosome Machinery Is Impaired in Atrogin-1 Deficient Zebrafish Embryos

**Figure 5:**
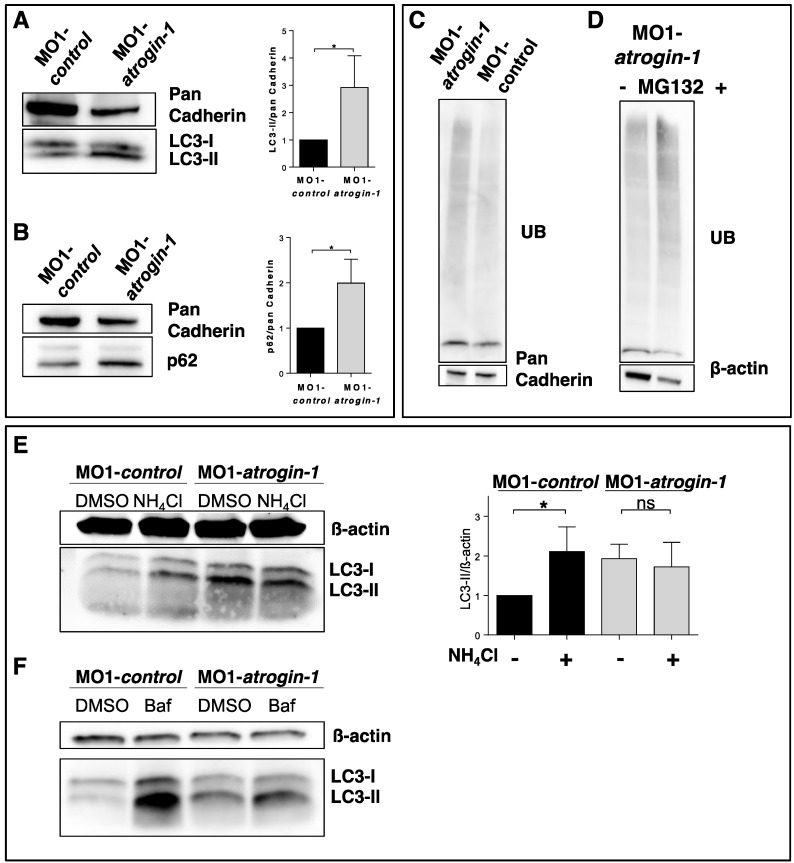
Atrogin-1 deficiency results in an accumulation of p62 and LC3-II and blocks autophagy. (**A**,**B**) Representative Western Blots of protein levels of the autophagy markers LC3 (**A**) and p62 (**B**) in MO1-*control* and MO1-*atrogin-1* injected embryos. Gray values were measured, quantified and data statistically evaluated (data represent means ± SD, student´s *t*-test, * p < 0.05) (**A**,**B**); (**C**,**D**) Western Blotting of ubiquitinated proteins in protein lysates of control injected embryos compared to Atrogin-1-deficient embryos (**C**) and Atrogin-1 morphants with or without treatment with the UPS inhibitor MG132 (**D**); (**E**) Western Blot analysis of MO1*-control* and MO1-*atrogin-1* injected embryos, DMSO or NH_4_Cl incubated for 12 h. Significant increase in MO1-*control* treated embryos compared to DMSO control, whereas no alteration was observed in MO1-*atrogin-1* morphants, data represent means ± SD, student´s *t*-test, * *p* < 0.05, ns = not significant; (**F**) Western Blot analysis of Bafilomycin A1 treated embryos.

Very recently, Atrogin-1 was shown to be critically involved in the regulation of autophagy in murine heart muscle tissue and that Atrogin-1 deficiency results in cardiomyopathy and heart failure due to autophagy impairment [14]. Hence, to assess whether Atrogin-1 deficiency also impacts on autophagy in zebrafish we evaluated the protein levels of p62 and LC3-II, both established markers of autophagy function. We found significantly increased levels of p62 and LC3-II in Atrogin-1 morphants compared to control-injected embryos (Figure 5A,B), a finding that is consistent with impaired autophagy. To test whether this increase is due to an induction of the autophagy/lysosome degradation system or a blockage of the autophagy MO1-*control* and MO1-*atrogin-1* injected embryos were treated with inhibitors of lysosome-autophagosome fusion (ammonium chloride (NH_4_Cl) and Bafilomycin A1 (Baf)). MO1-*control* embryos treated with NH_4_Cl demonstrated a significant increase in LC3-II levels in comparison to the DMSO treated MO1-*control* embryos (Figure 5E). Interestingly, we found no significant alteration in LC3-II levels in MO1-*atrogin-1* morphants treated with NH_4_Cl in comparison to their DMSO treated littermates (Figure 5E). Studies with the second lysosome-autophagosome fusion inhibitor Bafilomycin A1 are in line with the findings of the NH_4_Cl treatment, revealing only a slight increase of LC3-II protein levels in MO1-*atrogin-1* morphants (Figure 5F). Together these findings indicate that a deficiency of Atrogin-1 leads to a block of the autophagy degradation pathway. Since both protein degradation systems, the autophagy/lysosome and Ubiquitin proteasome system, are possibly functionally interconnected, we next analyzed the amount of ubiquitinated proteins in Atrogin-1 morphants and control-injected embryos to assess UPS function *in vivo*. We found that levels of ubiquitinated proteins are increased in Atrogin-1 morphants compared to controls (Figure 5C), suggesting that in addition to impaired autophagy also the UPS might be compromised due to the loss of Atrogin-1. To test this presumption, we knocked down Atrogin-1 and subsequently incubated these zebrafish embryos with the established UPS inhibitor MG132 for 24 h. Surprisingly, we found that after MG132 treatment levels of ubiquitinated proteins were further increased (Figure 5D), implying that the UPS per se is functional in Atrogin-1-deficient zebrafish embryos. However we cannot exclude the possibility that the increase of UB-proteins is due to an accumulation of p62, which can cause a decreased clearance of UB-proteins [17].

### 2.4. Atrogin-1 Deficiency Leads to an Autophagy-Related Ultrastructural Muscle Pathology

**Figure 6:**
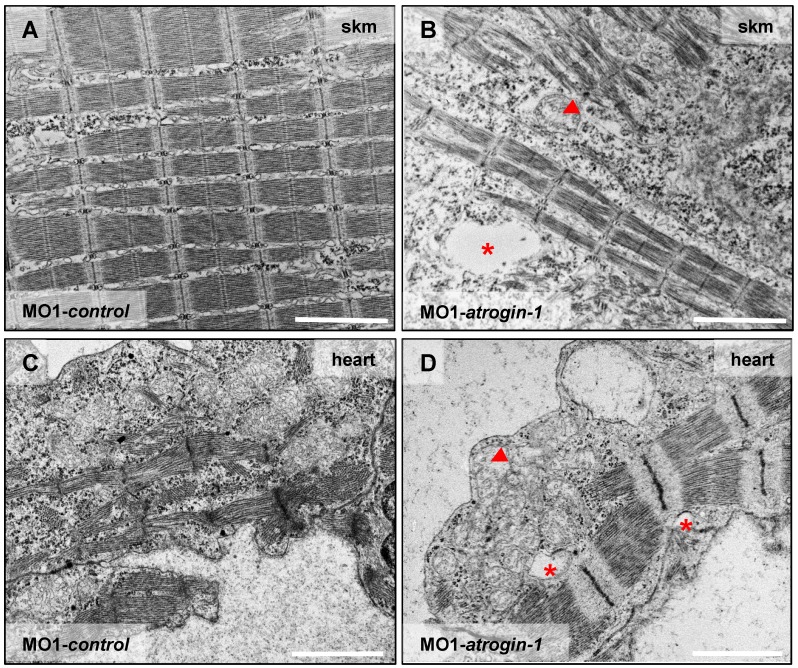
Atrogin-1 deficiency leads to an autophagy-related ultrastructural muscle pathology. (**A**–**D**) Electron micrographs of cardiomyocytes (heart) and skeletal muscle cells (skm) of Atrogin-1 morphants and embryos injected with control Morpholino at 72 hpf. Asterisks indicate vesicular bodies, arrowhead indicates dysmorphic mitochondria, scale bar = 2 µm.

Impaired autophagy in muscle cells is known to trigger severe ultrastructural alterations often leading to compromised muscle function and ultimately heart failure [14]. To investigate whether impaired heart and skeletal muscle function in Atrogin-1 morphant embryos is due to a defective ultrastructural architecture, we performed transmission electron microscopic analyses of heart and skeletal muscle tissue. Skeletal muscle cells of control-injected embryos were densely packed with sarcomeres, which are composed of highly organized arrays of thick and thin filaments, flanked by well-defined Z-disks (Figure 6A, for higher resolution pictures see Figure S1H,I). Cell nuclei are of elongated appearance and mitochondria are composed of numerous cristae in control muscle tissues (Figure S1F,H). By contrast, Atrogin-1 morphant muscle showed severely disorganized and pinched out myofibrils and sarcomeres as well as rounded muscle cell nuclei (Figure 6B and Figure S1G,I). Furthermore, Atrogin-1 morphants exhibited dysmorphic mitochondria with severely reduced numbers of cristae (Figure 6B arrowhead). In addition to these pathologic features, Atrogin-1-deficient skeletal muscle showed an accumulation of vesicular bodies (Figure 6B asterisk), compared to control-injected embryos (Figure 6A). Intriguingly, ultrastructural analyses of cardiomyocytes of Atrogin-1 morphant embryos revealed similar ultrastructural alterations as observed in Atrogin-1-deficient skeletal muscle cells, including the reduction of organized sarcomeres and the accumulation of pathological vesicular bodies (Figure 6D), compared to control-injected embryos (Figure 6C).

In summary, these findings indicate that loss of Atrogin-1 in zebrafish leads to pathologically altered muscle ultrastructure causing heart failure and skeletal muscle dysfunction *in vivo*.

## 3. Discussion

Fine-tuned protein turnover by the *de novo* synthesis and degradation of proteins is fundamental to ensure almost all cellular functions. Particularly, proper function of the muscular compartment including myocardium and skeletal muscle strongly depends on undisturbed protein homeostasis. In this context, impaired proteostasis was demonstrated to cause severe cellular defects often leading to heart failure and myopathies [18,19]. Particularly, defective degradation and removal of damaged and un-/misfolded proteins are detrimental for the proper function of the heart and skeletal muscle [6,18,19]. Protein degradation is essentially accomplished by the autophagy/lysosome system and the Ubiquitin proteasome degradation system (UPS). Both cooperative proteolytic systems are essential to control protein quality and quantity *in vivo*. However, the molecular mechanisms that mediate the tight regulation of protein degradation are largely unknown. Here, we characterized the *in vivo* role of Atrogin-1 in the vertebrate model system zebrafish and found that Atrogin-1 deficiency leads to heart and skeletal muscle dysfunction likely due to autophagy insufficiency.

In mammals, Atrogin-1 (MAFbx; FbxO32), an E3 ubiquitin ligase exclusively expressed in the heart and skeletal muscle, was identified as an important mediator of muscle atrophy [11,15]. Targeted overexpression of Atrogin-1 in myotubes leads to severe atrophy, whereas Atrogin-1 ablation in mice protected against atrophy [11]. Similar observations were made after Atrogin-1 induction in the heart [13,20,21], suggesting that Atrogin-1 controls muscle mass in both the heart and skeletal muscle under physiological and pathophysiological conditions. We found that Atrogin-1, similar to the situation in mice and humans, is strongly expressed in the zebrafish skeletal muscle and heart, suggesting evolutionarily conserved biological functions between zebrafish and mice/humans [11]. Remarkably, Atrogin-1 knockout mice are viable and fertile and the skeletal as well as cardiac muscle appears unaffected by the loss of Atrogin-1 under physiological conditions until the age of nine months [11,14]. By contrast, we found that targeted inactivation of zebrafish Atrogin-1 results in a severe and progressive impairment of heart and skeletal muscle function starting as early as 48 h post fertilization, suggesting that zebrafish Atrogin-1 facilitates additional developmental functions or that other E3 ubiquitin ligases can compensate for the loss of Atrogin-1 in mouse muscle during the first nine months after birth. As demonstrated by Sandri and coworkers, aged Atrogin-1 knockout mice (>9 months) develop severe cardiomyopathy and premature death due to defective autophagy [14]. Similarly, also muscle function and force generation is greatly reduced in adult Atrogin-1 knockout mice [22]. Ultrastructural analyses of these knockout mice revealed severe ultrastructural abnormalities such as disappearance of sarcomeres, the basic contractile units, and abnormal mitochondria, both features consistent with impaired autophagy [14]. Interestingly, in Atrogin-1 morphants, similar to the situation in Atrogin-1 null mice, sarcomeres are also disassembled and pinched out, mitochondria are dysmorphic and vesicular bodies are present. Originally, Atrogin-1 was identified to be involved in the degradation and removal of Calcineurin as well as the regulation of Forkhead box (FoxO) transcription factors [12,20,21,23,24]. Very recently, Zaglia *et al.* [14] found that mice lacking Atrogin-1 display defective degradation of CHMP2B (charged multivesicular body protein 2 B), a part of the endosomal sorting complex (ESCRT) that is crucial for autophagy function. In aged Atrogin-1 knockout mouse hearts, CHMP2B turnover is diminished leading to its intracellular accumulation/aggregation, impairment of autophagy and finally proteotoxic ultrastructural alterations in cardiomyocytes and heart failure [14], highlighting the importance of orchestrated protein degradation in muscle cells to prevent heart and skeletal muscle damage.

In Atrogin-1 morphant zebrafish embryos, we find altered autophagy as early as 48 hpf, described by the accumulation of the autophagic markers p62 and LC3-II. To determine whether this increase of p62 and LC3-II protein levels is due to a more active state or an inhibition of the autophagy/lysosome system, we measured changes in autophagic flux in MO1-*control* and MO1-*atrogin-1* injected zebrafish embryos after incubation with the established lysosome-autophagosome fusion inhibitors ammonium chloride (NH_4_Cl) or Bafilomycin A1 (Baf) [25,26,27]. The treatment was expected to increase LC3-II levels in MO1-*control* injected embryos since blocked lysosome-autophagosome fusion disturbs regular lysosomal degradation of LC3-II, leading to an accumulation of LC3-II. Indeed, we find a significant increase in LC3-II levels in MO1-*control* embryos treated with NH_4_Cl or Bafilomycin A1 in comparison to DMSO-treated MO1-*control* embryos. By contrast, in MO1-*atrogin-1* morphants, only a mild increase or even no effect on LC3-II protein levels were expected if the loss of Atrogin-1 leads to an impairment of autophagic flux. In fact, we find that LC3-II protein levels do not significantly accumulate further in Atrogin-1 morphants treated with either ammonium chloride (NH_4_Cl) or Bafilomycin A1 (Baf), suggesting that autophagy is indeed blocked in Atrogin-1-deficient zebrafish embryos. Additionally, we investigated the levels of ubiquitinated proteins and found their levels increased in Atrogin-1-deficient embryos compared to control embryos. This might be caused by an inhibition of the UPS or the autophagy/lysosome system, since both degradation pathways degrade ubiquitinated proteins. Interestingly, Korolchuk and coworkers [17] were able to demonstrate that the inhibition of autophagy severely increases the levels of Ubiquitin-Proteasome substrates and that this increase is largely due to the accumulation of p62 proteins which subsequently inhibits the clearance of ubiquitinated proteins destined for proteasomal degradation. As mentioned above, we find an increase of ubiquitinated proteins in Atrogin-1 morphant zebrafish. After treatment of Atrogin-1 deficient zebrafish with MG132, a highly effective inhibitor of the UPS, levels of ubiquitinated proteins are further increased, suggesting that the UPS per se might be still functional. Nevertheless, MG132 was also shown to induce the autophagy system which can subsequently result in an increase of ubiquitinated proteins. Whether the observed increase of ubiquitinated proteins in Atrogin-1 morphants is due to the accumulation of p62 remains unknown.

Impaired autophagy, as observed in Atrogin-1 knockout mice or morphant zebrafish, is found in various heart and skeletal muscle disease models [7,28,29,30,31,32,33]. The pathophysiological relevance of the autophagy/lysosome system in myofibrillar myopathies (MFM) is emphasized by Tannous and coworkers that show that the impairment of autophagic processes in a transgenic mouse model expressing an αB-crystallin mutant (CryABR120G) leads to a dramatic increase in the severity of the muscular pathology [34,35]. Furthermore, cardiac-specific ablation of Atg5, critical for autophagy function, causes heart failure in aged mice [36], whereas the accumulation of misfolded mutant cardiac Myosin binding protein-C (cMyBP-C) impairs protein homeostasis and causes cardiomyopathies in mice and humans [7,37].

These findings highlight the fundamental role of orchestrated protein homeostasis, in particular of protein degradation and removal, for the proper function of the heart and skeletal muscle. Here, Atrogin-1 seems to play an important role in fine-tuning protein degradation since its deficiency leads to cardiomyopathy and skeletal muscle disease, thereby introducing Atrogin-1 as a potential therapeutic target to modulate muscle disease caused by proteotoxic mechanisms. In this context, the zebrafish has emerged as an important vertebrate animal system to model and study human muscle diseases such as cardiomyopathies or myofibrillar myopathies [16,38,39] as well as for *in vivo* drug discovery using high-throughput small compound screening platforms (SCS) [40,41,42,43]. In contrast to the situation in Atrogin-1 knockout mice that develop heart failure at the age of nine months at the earliest, Atrogin-1 morphant zebrafish display severe cardiac and skeletal muscle dysfunction due to impaired autophagy as early as two days post fertilization, demonstrating that embryonic zebrafish deficient for Atrogin-1 might be (1) a suitable vertebrate model to study Atrogin-1-mediated autophagic muscle pathologies and (2) an elegant and valuable *in vivo* bioassay to identify and characterize novel therapeutically active substances for the modulation of disease pathologies related to defective protein homeostasis.

## 4. Materials and Methods

### 4.1. Zebrafish Strains

Care and breeding of zebrafish *Danio rerio* was carried out as described [44]. The present study was performed after appropriate institutional approvals. It conforms to European Union Directive 2010/63/EU. Embryos were staged as described previously in hours post fertilization (hpf) [45]. Pictures and movies were recorded at 16 and 18 somites stage and 24, 48, and 72 h post fertilization. For pigmentation inhibition, zebrafish embryos were treated with 0.003% 1-phenyl-2-thiourea.

### 4.2. Morpholino and mRNA Injection Procedures

Morpholino-modified antisense oligonucleotides (MOs; Gene Tools, LLC 1001 Summerton Way, Philomath, OR 97370, USA) were directed either against the translational start site of zebrafish atrogin-1 (MO1-*atrogin-1*: 5′-GTCTTGTCCAAGAAACGGCATTGTC-3′) or the splice-donor site of exon 1 (MO2-*atrogin-1*: 5´-AGTGCAGATTAAACACCGACCTCTT-3′) with the appropriate five base pair mismatch MOs (MO1-*control*: 5′-GTATTGTACAACAAACGCCATTCTC-3′ and MO2-*control*: 5′-AGTCCACATAAAACAGCCACCTCTT-3′). Morpholinos were injected into one-cell stage embryos with 5.4 ng MO1-*atrogin-1*, MO1-*control* and 7.2 ng MO2-*atrogin-1*, MO2-*control* in 0.2 M potassium chloride. For rescue experiments, sense-capped mRNA of myc-tagged *atrogin-1* was synthesized using the mMESSAGE mMASCHINE system (Ambion, Cat.No. AM1340). All injections were performed into one-cell zebrafish embryos.

### 4.3. Isolation of Embryonic Zebrafish Hearts and Immunostaining

Embryos were fixed in 4% paraformaldehyde for immunohistological stainings, RNA *in situ* hybridization and hematoxylin and eosin stainings. Immunohistological stainings were carried out with antibodies against Atrogin-1 (1:200, ECM Bioscience; polyclonal rabbit AP2041, Versailles, KY 40383, USA) and ß-catenin (1:200, Abcam ab6301, Cambridge, CB4 0FL, UK). Hearts were dissected at 72 hpf and immunofluorescent staining was performed as described [46]. Nuclei were counterstained with DAPI.

### 4.4. Zebrafish Protein Lysate Extraction and Western Blot Analysis

Protein extraction of embryonic zebrafish and immunoblotting was performed as described [47] using following primary antibodies: anti-Atrogin-1 (1:1000), mouse anti-ubiquitin (P4D1) (1:1000, cell signaling #3936S, Danvers, MA 01923, USA) monoclonal rabbit anti-p62 (1:2000, Abcam ab109012), rabbit anti-LC3 (1:2000, novusbio NB100-2331) and mouse anti-Myc (1:10000; cell signaling #2276S). For loading control mouse anti-β-actin (1:1000; Sigma-Aldrich #A5441) and rabbit anti- pan-cadherin (1:50000; Abcam #ab16505, 330 Cambridge, CB4 0FL, UK) were used. Signals were detected by chemiluminescence (anti-mouse IgG HRP-linked, anti-rabbit IgG HRP-linked, Cell signaling #7076/#7074) using a Luminescent image analyzer (Image Quant LAS4000mini, D-79111 Freiburg, Germany). Protein levels were compared by measuring the mean gray values, LC3-II and p62 protein levels of MO1-*control* injected embryos were normalized to 1.

### 4.5. Ammonium Chloride, Bafilomycin A1, MG132 Treatment

Forty eight hpf zebrafish embryos were treated with 100 mM ammonium chloride (Merck) in E3 for 4 h or with 20 nM DMSO-dissolved Bafilomycin (Sigma-Aldrich, Sigma-Aldrich Corp., St. Louis, MO, USA) in E3 for 12 h [27]. 24 hpf control and Morpholino injected embryos were treated with 100 µM MG132 (Tocris Bioscience, Bristol, BS11 9QD, UK) in E3 for 24 h.

### 4.6. Reverse Transcriptase (RT)-PCR and RNA in Situ Hybridization

RNA isolation was performed by using Qiazol Lysis Reagent (Qiagen, Hilden, Germany), according to the manufacturer’s instructions. cDNA synthesis was carried out by using 1 µg total RNA, oligo(dT) primer and SuperScript^®^ III Reverse Transcriptase (Life Technologies, Carlsbad, CA, USA). RT-PCR was performed according to standard protocols with *atrogin-1* specific primers. RNA whole-mount *in situ* hybridization using *myoD*, *myoG* (*myogenin*), *atrial myosin heavy chain* (*amhc; myh6*) and *ventricular myosin heavy chain* (*vmhc*) probes was carried out as described [48].

### 4.7. Birefringence, Spontaneous Movement, and Touch-Evoke Escape Response Assay

The noninvasive birefringence and touch-evoke escape response analysis was carried out and analyzed as described [16]. The movement after tactile stimulation was classified in three groups: adequate, inadequate, and no movement. For the spontaneous movement assay false-colored superimposed overviews of 24 hpf embryos were analyzed.

### 4.8. Histology and Transmission Electron Microscopy

Fixed embryos were embedded in JB-4 (Polysciences, Inc., Warrington, PA, USA) and 5 µm sections were cut, dried and stained with hematoxylin/eosin [49]. Histological analysis and transmission electron micrographs of embryos were carried out as described [16].

### 4.9. Functional Assessment and Statistical Analysis

Images were taken with an Olympus SZX 16 microscope and movies were recorded with a Leica DM IL LED microscope (Leica Mikrosysteme Vertrieb GmbH, Wetzlar, Germany). The functional assessment of cardiac contractility was carried out as described [49]. Fractional shortening and ventricular diameters were measured with the help of the zebraFS software [49] If not further specified, results are expressed as mean ± SD. Analyses were performed using unpaired Student’s *t*-test and a value of *p* < 0.05 was accepted as statistically significant.

## Supplementary Materials

Supplementary materials can be found at http://www.mdpi.com/1422-0067/17/2/187/s1.
