# Supplementary material for: Atrogin-1 Deficiency Leads to Myopathy and Heart Failure in Zebrafish
Source: Int J Mol Sci. 2016 Jan 30;17(2):187. doi: 10.3390/ijms17020187 (PMC4783921; doi:10.3390/ijms17020187)
Supplement: Supplementary file 1 [file ijms-17-00187-s001.zip › ijms-110615-Supplementary Materials/Figure S1.pdf]

Supplementary Materials:Atrogin-1 Deficiency Leads to Myopathy and Heart Failure in Zebrafish

Anja Bühler, Monika Kustermann, Tiziana Bummer, Wolfgang Rottbauer, Marco Sandri and Steffen Just

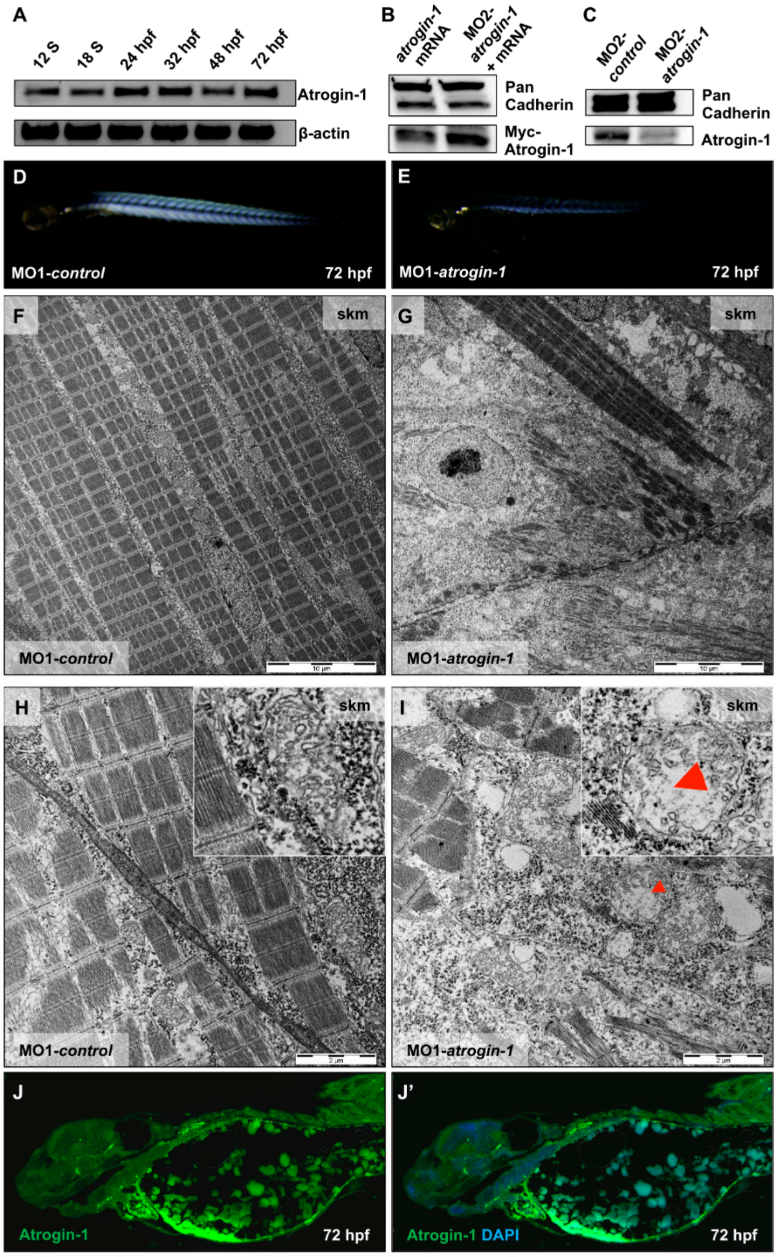

Figure S1. (**A**) RT-PCR for atrogin-1 and ß-actin on wild-type on zebrafish cDNA from 12-Somite stage (S) until 72 hpf; (**B**) Western blot analysis using an anti-Myc antibody after atrogin-1 mRNA and MO2-atrogin-1 + atrogin-1 mRNA injection; (**C**) Immunoblot analysis using an anti-Atrogin-1 antibody after MO1-control and MO2-atrogin-1 injection; (**D**,**E**) Lateral views of control injected (**D**) and MO1-atrogin-1 injected embryos (**E**) showing birefringence at 72 hpf; (**F**–**I**) Electron micrographs of skeletal muscle cells (skm) of Atrogin-1 morphants (**G**,**I**) and embryos injected with control Morpholino (**F**,**H**) at 72 hpf. (**J**,**J'**) Immunofluorescent staining against Atrogin-1 on paraffin sections, in lateral view of wild-type embryo at 72 hpf, nuclei are counterstained with DAPI.
